# Troubleshooting Pacemaker Behavior: Consequence of Prolonged Ventriculoatrial Conduction

**DOI:** 10.19102/icrm.2020.110905

**Published:** 2020-09-15

**Authors:** Sumer K. Dhir, Grant E. Gould, John L. Joliff

**Affiliations:** ^1^The University of Kansas Physicians, Heart and Vascular Center, Topeka, KS, USA; ^2^The University of Kansas Health System, St. Francis Campus, Topeka, KS, USA; ^3^Biosense Webster, Diamond Bar, CA, USA

**Keywords:** cardiac pacemaker, implantable cardioverter-defibrillator, pacemaker-mediated tachycardia, repetitive nonreentrant ventriculoatrial synchrony, retrograde conduction

## Abstract

The event of repetitive nonreentrant ventriculoatrial synchrony (RNRVAS) and its course of cardiac device management has been authenticated. However, this context has not been well-documented in the presence of high-degree antegrade heart block. This case report will discuss the challenges of treatment in this subgroup of patients.

## Case presentation

**Figure 1:**
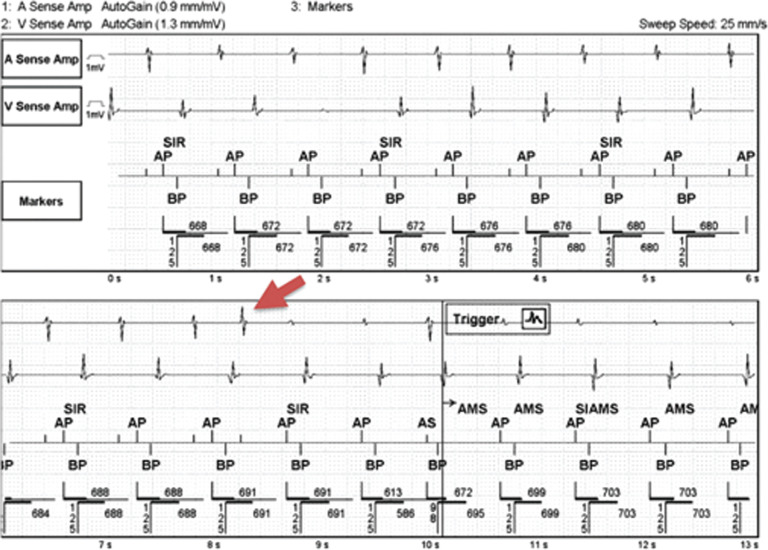
Device interrogation of atrial and ventricular electrograms. AP: atrial pacing; BP: biventricular pacing; AMS: automatic mode switch; SIR: sensor-indicated rate.

A 55-year-old male presented with a complex cardiac history including the implantation of a cardiac resynchronization therapy (CRT) defibrillator for symptomatic second-degree atrioventricular (AV) block type II and cardiomyopathy. In the past, the patient had demonstrated pacemaker-mediated tachycardia (PMT), despite his antegrade conduction disease, for which the device’s postventricular atrial refractory period (PVARP) was extended to treat the arrhythmia successfully. The patient later presented to the emergency department with complaints of palpations and dizziness. The patient’s device was interrogated **(Figure 1)** and revealed repetitive nonreentrant ventriculoatrial synchrony (RNRVAS), also known as atrial ventricular desynchronization arrhythmia.^1^

## Discussion

RNRVAS is closely correlated with PMT; however, it is less often observed in the presence of high-degree AV block.^2^ The presence of retrograde VA conduction is a required substrate for these types of arrhythmias and any AV desynchronization coupled with VA conduction may have the potential to initiate both.^1^ Both arrhythmias prompt similar symptoms due to the interference of atrial function causing neurocardiogenic effects. A differentiating feature is that RNRVAS more often occurs at slower rates, while PMT occurs at or near the device’s maximum tracking rate.^2^

**Figure 2:**
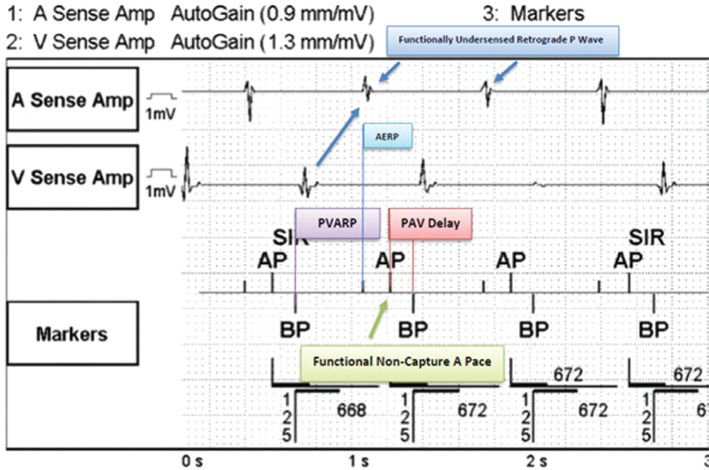
The sequence of RNRVAS. AERP: atrial effective refractory period; PAV: paced atrioventicular; PVARP: post Ventricular Atrial Refractory Period.

RNRVAS is often initiated by a premature ventricular contraction (PVC) that conducts retrogradely to the atria. Subsequently, the retrograde P-wave falls into the device’s PVARP and, in turn, is functionally undersensed. This means that the device will not respond to the retrograde activation and, because of the relatively long atrial effective refractory period, the device delivers a functional noncaptured atrial pacing impulse. At this point, the device reaches the paced AV delay limit, generating a ventricular pacing impulse that again propagates retrogradely to the atria via the intrinsic VA conduction, prompting the endless loop phenomenon^3,4^ **(Figure 2)**.

**Figure 3:**
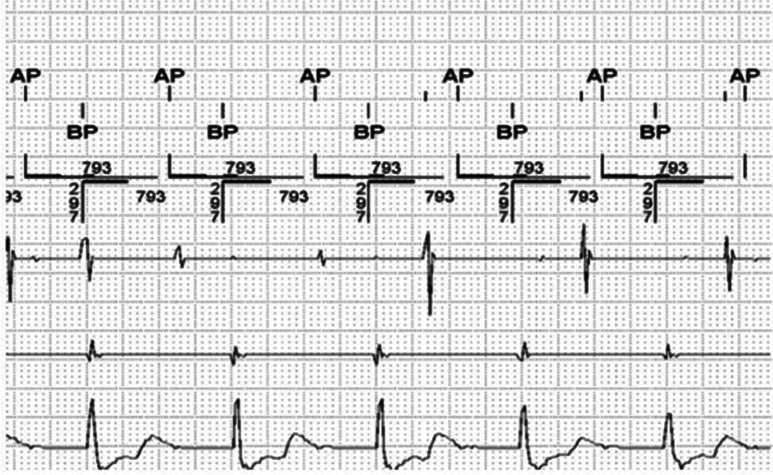
The initiation of RNRVAS with a biventricular pacing event. It can be seen that the retrograde atrial depolarization falls into PVARP. The PVARP parameter was set to 450 ms in this instance to prevent PMT. Thereafter, atrial pacing occurred and was classified as functional noncapture as the atrium had just depolarized.

However, an interesting aspect in the current case was that RNRVAS was provoked by prolonged VA conduction rather than a PVC. As shown in **Figure 3**, the initiation of the phenomenon was begun with biventricular pacing that propagated retrogradely to the atrium with a prolonged conduction time of 426 ms.

Referring again to **Figure 1**, the red arrow could demonstrate a premature atrial depolarization that disrupts VA conduction and consequently terminates the rhythm by concealed infiltration of the AV node.^5^ Retrograde dual AV nodal physiology must also be considered. In that instance, such possibly makes the atrial event at the red arrow a retrograde P-wave propagating over the fast-retrograde pathway. Of note, this beat occurs sooner and increases the device’s atrial escape interval (AEI).

There have been multiple programming modifications described to circumvent RNRVAS. A case study proposed that setting the rate-response PVARP to the shortest minimum duration may interrupt and help avoid sensor-driven RNRVAS.^6^ Other programming adjustments, such as shortening the paced AV delay and/or decreasing the lower pacing rate, should be considered.^7^ To our knowledge, there are currently no existing device algorithms to avoid, recognize, or even terminate RNRVAS.^8^

Initially, more specific individualized programming modifications were attempted due to the patient’s prior history of PMT and the unusual initiation of RNRVAS. It was our goal to increase the atrial rate and promote atrial activation antegradely before the impulse could be propagated in the retrograde direction. Thus, the paced and sensed AV delays were extended to 300 ms and 275 ms and the base rate was increased to 85 bpm (705 ms). With these parameters increased, the device was expected to pace the atrium at the base rate of 705 ms and before the retrograde impulse would propagate to the atrium (ie, 300 ms paced AV delay plus a VA conduction time of 426 ms equals a total of 726 ms). These parameter changes were successful in the interim by suppressing the VA conduction and eliminating the RNRVAS by essentially reconstructing the concealed incomplete infiltration of the AV node as the premature atrial contraction did. However, the patient did not tolerate the increased base heart rate and more conventional device programming steps were adopted to resolve the matter, including decreasing the paced AV delay to 125 ms and 100 ms and decreasing the lower pacing rate to 50 bpm. By decreasing these parameters, the functional noncaptured atrial pacing impulse was delayed and the AEI was increased.^6^ The patient’s CRT device was programmed to the DDDR mode with an maximum tracking rate of 105 bpm. As seen in **Figure 1**, the device’s sensor was active and contributed to RNRVAS by decreasing the AEI, the opposite of what was just explained. We considered eliminating the patient’s sensor; however, ultimately, we left it as turned on due to the patient’s active nature but reduced its sensitivity.

Prior studies looking at the presence of intact VA conduction with complete heart block have reported a prevalence of 14% to 15%.^9,^^10^ In our recent study looking at 47 patients with a cardiac rhythm management device and complete heart block, 12% of patients demonstrated intact retrograde VA conduction.^11^ Despite antegrade conduction disease, patients can show retrograde VA conduction, resulting in device-mediated arrhythmias.

The patient was seen for follow-up a couple months later without the observation of any significant arrhythmias.

## Conclusion

This report exemplifies device-mediated arrhythmias and the challenges faced in such groups of patients. Understanding the mechanism of pacemaker-mediated arrhythmias is important for programming and treating these patients.
